# Supplementary material for: Are carbon emissions trading and green financial instruments synergistic? -Comprehensive quantitative research based on content analysis
Source: PLoS One. 2024 Mar 7;19(3):e0298601. doi: 10.1371/journal.pone.0298601 (PMC10919601; doi:10.1371/journal.pone.0298601)
Supplement: S1 Data — (ZIP) [file pone.0298601.s001.zip › Data/地级市Z_F工作报告.zip!/╝¬┴╓/░╫│╟╩╨/└┤╫╘╣1⁄2╓┌║┼í╛┬φ┐╦╩2╛▌═°í┐_2012.docx]

各位代表：

　　现在，我代表白城市人民政府，向大会报告政府工作，请予审议，并请市政协各位委员提出意见。

　　一、2011年工作回顾

　　2011年，是“十二五”开局之年，也是我市蓄势赶超谋发展、砥砺奋进促跨越的一年。在省委、省政府和市委的正确领导下，在市人大、市政协的监督和支持下，我们坚持以邓小平理论和“三个代表”重要思想为指导，深入贯彻落实科学发展观，紧紧围绕建设吉林西部特色经济区，全力构建“五大基地”，加快开发区“二次创业”，全市呈现出经济增长、民生改善、社会和谐的良好局面，较好完成了市四届人大四次会议审议确定的目标任务，实现了“十二五”良好开局。

　　全市地区生产总值预计完成530亿元，增长19%，增速位居全省前列。规模以上工业增加值预计完成120亿元，增长34.4%，增速位居全省第一。固定资产投资预计完成355亿元，增长30.5%。财政收入再上新台阶，全口径财政收入完成42.5亿元，增长29.5%。地方级财政收入完成24.6亿元，增长36.4%。粮食产量创历史最好水平，增长26.2%。预计城镇居民人均可支配收入18003元，农民人均纯收入5500元，分别增长13.2%和22.1%。这些成就取得，标志着我们开创了加速崛起、富民强市的崭新局面，迈出了后发赶超、跨越发展的坚实步伐，增强了稳中求进、好中求快的信心和决心。

　　一年来，我们重点抓了以下工作：

　　（一）全面实施“五大基地”建设攻坚。突出投资拉动、项目带动，全市3000万元以上项目发展到700个。新开工建设项目146个，超亿元项目76个。引进资金460亿元，增长33%。大项目带动、集群式发展成效凸显。能源基地建设取得新进展。新开工建设风电场10个，全市发展到37个，装机突破300万千瓦，增长40.8%；并网达200万千瓦，增长22.7%。风电设备制造企业发展到15户，我市已成为全省最大的风电设备配套中心。新能源装备制造园区被省政府评为特色园区。通榆禾佳稻壳发电、国电龙华一期2×20万千瓦热电联产扩建项目分别竣工和投产。大安天威光伏发电、洮南大唐三合“金太阳”示范工程等项目加快推进。油气当量达到186万吨。全市形成了“清洁能源优先，风电火电并重，多种能源互补”的开发局面。粮食生产基地建设有了新成效。加快粮食增产工程建设，“引嫩入白”、大安灌区主体工程已完工并发挥效益。洮儿河灌区续建配套工程加速推进。旱田高效节水灌溉工程加快实施，全程灌溉面积达到460万亩，膜下滴灌面积达到43.6万亩。粮食产量跨上了新的阶段性水平。加强菜篮子工程建设，全市蔬菜种植面积达到100.5万亩。特色农产品加工基地建设登上新台阶。坚持引龙头、建基地、打品牌，新开工建设益海嘉里葵花油生产、天兴蓖麻产业园等10个3000万元以上项目，建设镇赉万头原生态奶牛场。特色农产品加工企业发展到1168户，实现产值170亿元，增长54.5%。“通榆草原红牛肉”被国家评为地理标志保护产品。农业园区不断发展壮大。新农村建设扎实推进。区域商品集散地建设实现新发展。白城欧亚购物中心二期工程竣工运营。内陆港物流中心、东北商贸城等项目启动推进。杂粮杂豆出口示范基地纳入省级规划。服务业增加值预计完成196亿元，增长18%。社会消费品零售总额预计完成191亿元，增长17.8%；外贸进出口总额预计完成1.1亿美元，增长35%。区域商品集散地辐射作用正在显现。生态产业基地建设迈出新步伐。学习长治、植树造绿，“举市三年大造林”完成41万亩，占年度任务的117%。我市被国家评为国土绿化突出贡献单位，被省政府评为十年绿化美化吉林大地先进市。百万亩芦苇湿地保护项目启动实施。大力发展生态旅游业，打造“湿地风光、鹤乡白城”特色品牌。全市旅游业收入完成19.5亿元，增长29.7%。全市生态产业发展步伐加快，整体效益不断提升。

　　（二）着力促进开发区大发展快发展。坚持内涵提升、外延驱动，加快开发区“二次创业”步伐。扩区建设加速启动。白城工业园区投资1.2亿元实施建设三期工程和综合配套服务区。白城经开区确定“六园一城”开发重点，投资1.2亿元新建、维修道路8条。查干浩特旅游经开区规划开发10个旅游景区。新建洮北经开区投资3亿元完成“七通一平”建设任务。各县（市）工业集中区配套建设加快推进，承载集聚功能明显增强。项目立区成效明显。各开发区引进3000万元以上项目126个，开工建设98个，续建项目56个，竣工投产21个，完成固定资产投资197.8亿元，增长68%。项目建设已成为开发区跨越发展的重要引擎。经济实力大幅跃升。全市开发区地区生产总值预计完成251亿元，增长53.5%；规模以上工业增加值预计完成105.5亿元，增长57%；全口径财政收入完成16亿元，增长51%。主要经济指标增幅都在50%以上，经济发展已步入了快车道，对全市引领带动作用不断增强。

　　（三）强力推进工业经济发展壮大。坚持引进增量、盘活存量、做大总量，全力实施工业倍增计划。生产性工业项目加快建设。开工建设171个项目，完成投资245亿元。争取国家、省级科技发展计划项目38项，实施科技发展计划项目137项，夯实了工业经济发展的基础。做大做强企业实现新突破。对正隆齿轮、通业机械等重点企业采取异地扩能改造、“退二进三”等措施，分企施策、盘活存量工作取得了明显成效。引进国药、中建材集团，推进医药、建材两大产业嫁接央企。全市清洁能源、油气化工、农产品加工、汽车配套、纺织医药、冶金建材、装备制造等七大产业，完成总产值370亿元，增长51%。培育规模企业取得有效进展。实施“中小企业百户成长型”工程，规模以上工业企业发展到285户。开展“金融服务年”活动，小贷公司已获准营业7家，担保公司获颁行政许可8家。加强上市后备企业培育，北方金塔、中一精锻、裕丰米业、艾倍特乳业、天合风电等16户企业取得积极进展。全市工业在总量扩张和能力提升上实现了历史性跨越。

　　（四）统筹推进特色城镇化发展。围绕特色城镇化建设，编制了全市城镇体系规划，启动实施新一轮城市总体规划。市区建设步伐加快。突出提升综合承载能力，完成了投资31.6亿元的10大工程、90个项目建设，开发建设23个住宅小区。围绕拉大城市发展框架，以南拓为主、西延跟进，加快推进新区开发建设前期工作。加强市容环境综合治理，以创建省级园林城、卫生城为目标，加大城市管理力度。依法整治营运秩序，打击非法营运车辆，城区交通秩序得到改善。实施“一桥、四场、五塔、两街、两路”亮化工程，市容市貌大为改观，城市形象明显提升。县城开发亮点纷呈。各县（市）围绕打造独具魅力的特色县城，掀起了大改造、大开发、大发展的热潮。大安完成“北部明珠”新城建设，洮南开发建设北部新城，镇赉启动建设100万平方米商住区，通榆墨宝园项目已竣工开园。岭下、坦途、万宝、瞻榆、安广等中心镇建设加快推进、成效彰显。积极破解交通瓶颈，重点加大长白快速铁路、白阿铁路扩能改造、嫩丹高速公路、长安机场等重点基础设施项目攻关推进力度，取得了重大进展。

　　（五）加大保障和改善民生力度。坚持民生优先，全力惠民生、办实事、增福祉。创业就业活动深入开展。全市城镇新增就业4.86万人，登记失业率3.9%，“零就业”家庭保持动态为零。社会保障体系不断完善。企业最低工资标准、退休人员基本养老金和城乡低保、城镇居民基本医疗保险、新型农村合作医疗等补助标准进一步提高。各项社会事业统筹发展。全面落实《中长期教育改革和发展规划纲要》。启动学前教育三年行动计划。实施校舍安全工程。新建农村标准化学校15所。全面推进医药卫生体制改革，所有乡镇卫生院、社区卫生服务机构实行基本药物零差率销售。稳步推进文化体制改革，乡镇综合文化站、农家书屋实现全覆盖。白城数字图书馆投入使用。开展了以建党90周年为主题的系列文体活动。深入开展双拥创建活动，为驻军家属实行货币补贴，我市在全省申报“全国双拥模范城”评选中名列前茅。低生育水平进一步稳定，幸福家庭建设工程稳步推进。民生民利实事较好完成。改造城市棚户区225.2万平方米，新建廉租房27.5万平方米，改造农村泥草房99082户。新建改造热力站20座，新增供热能力918.5万平方米，改造供热管网197.5公里，既有建筑节能改造288.2万平方米。“气化白城”规划启动实施。白城中心医院建设项目完成门诊楼浇筑封顶，白城中医院新建项目完成土地预审、选址、环评等前期工作。阳光休闲广场、市殡仪馆改造工程已竣工使用。全民健身中心开工建设。海明路步行街改造和地下人防工程主体已完工。建设农村公路631.2公里，基本实现了村村通等级公路。又有6.5万农村贫困人口实现脱贫，7.5万人口用上安全水。社会管理创新有效推进。深入开展“平安白城”建设，严厉打击各类违法犯罪活动，“清网行动”成效显著。加强社会治安巡逻防控体系建设，社会治安形势持续稳定。“六五”普法工作全面开展。高度重视信访工作，信访总量、进省访总量、进京访总量分别下降12%、28%和37%。积极办理行政复议案件，立案审理行政复议案件135件。深入开展安全生产责任落实年活动，事故起数、死亡人数分别下降38.8%和16.4%。集中开展食品药品安全专项整治行动，实现了无药害事件、无餐饮重大食品安全事故“双无”目标。积极做好物价稳定工作。强化应急管理，各类突发事件得到有效处置。

　　（六）切实推进服务型政府建设。实施阳光政务，努力提升行政效能。监督机制不断完善。建立办理人大代表建议、政协提案制度，自觉接受人大监督、法律监督和政协民主监督，办理人大代表建议81件、政协委员提案79件，办结率均达到100%。服务效能得到提升。全面实施“12345”服务工程，构建绿色服务通道，对179项审批项目建立电子监察系统，有力推进了行政效能提高。政风转变成效明显。深入开展政行风评议活动。全面实行“市长直通车”、市领导接访、网络问政等“六项制度”，取得了明显成效。在政府系统开展了“讲学习、讲效率、讲认真、讲创新、讲奉献”活动。深入推进反腐倡廉建设，加大源头治腐力度，深入探索“制度+科技”创新举措，加强重点领域纠风治理力度，营造了廉洁高效的政务环境。

各位代表：

　　过去的一年，是重任在肩、矛盾叠加、艰难爬坡的一年，是负重拼搏、凝心聚力、提速赶超的一年。成绩来之不易。这是市委总揽全局、正确领导的结果，是市人大、市政协有力监督、全力支持的结果，是全市广大干部群众戮力同心、拼搏奋斗的结果。在这里，我代表白城市人民政府，向全市广大工人、农民、知识分子和各级干部、离退休老同志，解放军驻白部队、武警官兵和公安干警，中省直各单位，表示衷心的感谢！向市人大、市政协、市法院、市检察院，各民主党派、工商联、人民团体和各界人士，表示衷心的感谢！向关心和支持白城发展的各界朋友，表示衷心的感谢！

　　回顾过去的一年，我们也清醒地认识到，全市经济社会发展还存在诸多问题。主要表现在超前谋划项目和招商引资力度不够，引进具有强力支撑和拉动作用的大项目偏少；城市建设和管理还存在许多薄弱环节，一些公共设施建设还相对滞后；城乡居民收入水平还比较低，特别是低收入群体生活还比较困难；维护稳定压力加大，社会管理还存在一些突出问题；政府自身建设有待进一步加强。对于这些问题，我们一定要高度重视，采取有力措施，认真加以解决。

　　二、2012年主要任务

　　刚刚胜利闭幕的市第五次党代会，是在“十二五”实现良好开局，白城发展步入关键时期召开的一次重要会议。大会高举科学发展的旗帜，站在吉林西部崛起振兴的高度，提出了今后五年乃至更长时期的发展思路、奋斗目标和主要任务，奏响了团结、奋进、赶超的主旋律，开启了白城富民强市、跨越发展的新征程。

　　学习贯彻市第五次党代会精神，是当前和今后一个时期全市上下首要的任务，要切实把广大干部群众的思想和行动统一到党代会决策部署上来，把智慧和力量凝聚到推动白城发展振兴的实践上来。当前，全市各级政府贯彻落实市第五次党代会精神，就是要立足以上带下，把精神状态全面调整好。从今天开始，从我做起，从手头的事儿干起，时不我待，自我加压，昂扬斗志，锐意进取。就是要注重科学谋划，把机遇资源充分利用好。善于从大局变换中把握发展机遇，从政策走向中寻求发展支点，从资源对接中增强发展优势，从扩大开放合作中提升发展层次。就是要逐级细化分解，把各项任务深入落实好。党代会报告提出的“三大战略”、“三化”统筹、“六大功能区”建设重要举措和“513”、“千百十”宏伟计划，要责任到人，细化到项目，分解到进度。白城改革发展稳定的重任要人人有责、事事落地、时时在推进。就是要加大攻坚力度，把突出难题尽快破解好。直面困难，主动出击，以改革的办法攻坚，以创新的举措突破，以克难的勇气化解，进一步拓展发展空间，优化发展环境。就是要狠抓制度创新，把投资环境切实建设好。转变政府职能，规范行政行为，提高服务水平，健全监督机制，营造良好氛围，努力打造企业愿意来、待得住、发展快的最佳投资环境。

　　今年，是实施“十二五”规划承上启下的重要一年，也是全面贯彻落实市第五次党代会精神的第一年，做好今年的工作意义重大。政府工作总的要求是：深入贯彻落实党的十七届六中全会、省委九届十二次全会、市第五次党代会精神，坚持以科学发展为主题、以转变发展方式为主线，围绕加快建设吉林西部特色经济区，大力实施“三大战略”，抓投资扩总量，抓转型调结构，抓改革破难题，抓民生促和谐，深入推进“五大基地”建设，加速开发区“二次创业”步伐，全力打造“六大功能区”，促进全市经济社会又快又好发展，以优异成绩迎接党的十八大和省十次党代会胜利召开。

　　全市经济社会发展主要预期目标是：地区生产总值完成620亿元，增长16.5%。规模以上工业增加值完成155亿元，增长27%。全口径财政收入完成51亿元，增长20%；地方级财政收入完成29.5亿元，增长20%。固定资产投资完成480亿元，增长36%以上。社会消费品零售总额完成225亿元，增长18.2%。城镇居民人均可支配收入20500元，增长13.9%；农民人均纯收入6000元，增长9%。

　　今年，我们要着力做好以下几方面工作：

　　（一）加速“兴工强市”进程，着力做大做强工业经济。推进新型工业经济主导区建设，全市工业总产值力争突破500亿元，新增规模以上企业要达到30户。

　　狠抓工业增量。坚持把生产性项目作为发展实体经济的重点，实施“513”发展计划，力争开工建设超10亿元项目50个以上、超亿元项目100个以上、超3000万元项目300个以上。抓好扶桑专用车生产等一批新开工项目，加快推进大连绿波甘草等一批续建项目，着力促进亿正药业、罗赛洛（大安）明胶等一批技改项目。生产性工业项目投资要达到265亿元，占全市固定资产投资的55%。

　　培育龙头企业。筛选100户关联度高、创新能力强、市场前景好的企业，通过上市融资、战略合作、兼并重组、品牌经营、技术改造及承接产业转移等措施，培育主营业务收入超10亿元、超50亿元、超百亿元的龙头型、基地型企业集团。加快做大益海嘉里、金事达电气、中一精锻、金升镍业、四季盛宝纺织、道君药业等龙头骨干企业。

　　推进全民创业。抢抓国家扶持小微企业发展契机，全面实施新一轮民营经济腾飞计划，坚持“抓大壮小扶微”，集中力量扶持100户“专、精、特、新”中小企业发展。培育5个优势产业集群，建设5个中小企业创业孵化基地。开展“万名创业者、万名小老板和万名技能工人”培训，强化创业服务指导，全年新增小企业270户，新增就业人员1.5万人以上。

　　（二）加快“二次创业”步伐，促进开发区高效集群发展。围绕把开发区建设成为解放思想的先导区、项目建设的集聚区、经济发展的领跑区和科学发展的示范区，全力实施招商兴区、项目立区战略。

　　加强项目攻坚。着力引进建设一批规模大的新项目、成长好的大项目、效益高的强项目。全市开发区年内必保落地50个亿元以上项目、新开工50个亿元以上项目，固定资产投资增幅达到50%以上，成为我市崛起振兴的重要增长极。

　　提升承载能力。按照组团发展、功能分区、滚动开发的原则，积极推进开发区的扩区、升级。白城工业园区要加速建设三期基础设施、综合配套服务区。白城经开区要加快基础设施配套建设。查干浩特旅游经开区要加快民族风情聚集区等10个旅游景区基础设施建设。洮北经开区要完善起步区配套服务体系，形成连接老城区的新兴产业聚集区。各县（市）要加大工业集中区基础设施建设力度，着力打造成县域经济跨越发展的重要平台。

　　推进产业聚集。加快城区企业“退二进三”、“退城进区”进程，推进汇锋汽车压铸件等企业入区扩能改造。启动建设新能源装备、汽配、纺织、医药、文化创意、新材料等产业园，做大做强装备制造、循环经济、特色农产品加工等产业园。各开发区地区生产总值、工业增加值、全口径财政收入增幅都要达到30%以上。

　　（三）突出清洁能源产业发展，加快推进能源基地建设。围绕创建国家清洁能源示范城市，坚持以风能、太阳能、生物质能开发为重点，发展壮大能源产业，产值力争实现153.6亿元。

　　抓好清洁能源开发利用。大力发展风电产业集群，推进洮南、通榆、大安等百万千瓦风电场开发，力争启动“安白”百公里新能源景观带二期工程，新增风电装机100万千瓦。建设太阳能发电基地，引进上海电气、中节能集团开发白城光伏发电示范区。加强洮南光伏产业园建设。积极争取国家和省政策支持，承接引进特种钢材、矿产冶金、环保建材等高载能项目，抓好清洁能源开发利用示范区。着力提高输变电能力，完成通榆500千伏输变电线路建设，争取洮南、镇赉、大安500千伏输变电工程早日开工。继续加大城乡电网改造力度。

　　加快火电热电项目建设。积极推进中电投白城发电公司二期2×100万千瓦火电工程、国电龙华白城热电厂二期2×35万千瓦热电联产扩建工程前期工作，争取尽早开工建设。抓紧启动通榆、大安、镇赉2×30万千瓦热电联产项目。

　　强化矿产资源勘探开发。加大油气开发力度，油气当量达到198万吨。推进“气化白城”工程。抓好万宝煤矿、聚宝铜矿等矿产资源开采。加强与大唐集团沟通对接，力争总投资210亿元的大型煤化工项目尽快启动。

　　（四）大力发展现代特色农业，开创“三农”工作新局面。紧紧围绕农业增产、农民增收、农村发展目标，加快特色精品农业拓展区建设。

　　提高粮食生产能力。抓住国家开展节水增粮行动机遇，加强旱田高效节水灌溉项目建设，全程灌溉面积达到520万亩，膜下滴灌面积累计发展到100万亩。加快建设吉林西部土地开发整理镇赉、大安项目区二期工程，加强土地后续平整耕种。建设旱涝保收高标准基本农田680万亩。扎实开展“农业农村经济科技效益年”活动，启动实施种子产业振兴、重大技术推广示范、全程农机化推进等工程。突出抓好36万亩保护性耕作技术推广，坚持依靠科技进步提高产量、提升质量、增加收益。

　　加快水利工程建设。抢抓国家水利改革发展机遇，打捆申报总投资267亿元的10个方面水利项目。启动分洪入向工程建设。加快中小河流治理、小型水库除险加固、灌区续建配套和节水改造等重点水利项目建设。完成月亮湖蓄滞洪区、洮儿河堤防工程可研批复工作，争取启动单项工程项目建设。加强水资源一体化管理，建立水利投融资体制机制。

　　发展特色效益农业。突出抓好水稻、绿豆、葵花、蓖麻、燕麦、奶牛、肉羊、鱼等特色农业，建设园区32个、百栋以上大棚园区10个、千亩以上种植园区10个、5万平方米以上牧业园区10个、20万平方米以上加工贸易园区2个。新建、改建标准化、规模化畜禽养殖场200个。实施“菜篮子”工程，建设10个蔬菜生产专业乡、8个棚膜蔬菜生产专业村，蔬菜种植面积达到90万亩以上。

　　推进产业化经营。启动建设3000万元以上农产品加工项目20个，其中超亿元项目5个以上。扶持一批农业产业化龙头企业做大做强，省级以上龙头企业发展到60户。农产品加工业总产值达到220亿元，增长29.4%。加强地理标志产品、特色农产品品牌培育，形成龙头带基地、基地带农户、农户带专业合作社的全链条、产业化发展格局。

　　加强新农村建设。加强农村水、电、路等基础设施建设，抓好农村危旧房改造、环境整治、饮水安全等工作，推进96个“千村示范、万村提升”省级示范村建设。

　　（五）强化城镇梯次开发集聚，加快城镇化发展步伐。坚持旧城提速改造与新区扩容开发并重，统筹推进城镇化发展，全市城镇化率力争达到42%。

　　开发新城区。着力建设功能齐全、布局合理、风格现代、结构优化、品质卓越、充满活力的现代化新城。坚持高标准规划、高水平设计、高质量建设，编制启动白城新城区总体规划和园林绿地、水系景观等专项规划。加快7平方公里的新城建设，开工建设“七通一平”基础设施，新城区主体框架基本形成。

　　改造旧城区。抓紧编制旧城区改造规划。市区投资21亿元，实施道路建设、供排水管网、亮化美化等8大工程60个项目，加快旧城区改造步伐。推进城市综合体建设，力争开工建设钻石?四季华城等项目，计划开发面积60万平方米。强化城市管理，探索建立有效城市管理体制机制。继续加强违法违章建筑整治、依法治理市区道路交通和营运秩序。深入开展创建活动，确保通过省级园林城、卫生城验收。

　　建设魅力县城。县（市）要立足资源禀赋、区位条件，加大县城基础设施建设力度，重点推进镇赉环南湖新城区，通榆东湖湿地，大安南湖新区，洮南关东古街开发改造、北部新城开发建设，不断提升县城品位。

　　做优中心镇。重点培育一批区位好、特色鲜明、有一定产业支撑和辐射带动功能的中心镇，着力推进镇赉坦途、洮南黑水、通榆瞻榆、大安安广、洮北林海等10个中心镇建设，强化公共服务设施建设，提高承载能力。

　　完善交通基础设施。重点开工建设长安机场、嫩丹高速公路、白阿铁路扩能改造等项目。启动建设金辉立交桥工程。加大长白快速铁路项目推进力度，努力构建以“两纵两横”高等级公路和干线铁路为支撑的交通网络。

　　（六）大力发展现代服务业，加强区域经济聚集中心区建设。实施服务业跨越发展计划，推动服务业发展提速。全市服务业增加值实现235.6亿元，增长18%。

　　建设服务业集聚区。加强小商品、现代物流、购物消费、特色农产品交易等服务体系建设。力争启动白城内陆港物流中心、向海物流中心、东北商贸城、综合物流产业园区等项目。完善洮南杂粮杂豆市场功能，力争镇赉粮食物流园区达到百万吨吞吐能力，着力培育一批大型服务业企业集团。

　　发展特色文化产业。抢抓中央深化文化体制改革的重大契机，坚持文化事业和文化产业两手抓，着力抓好规划布局，推进文化产业集聚区、产业带建设。规划建设文化项目35个，推进丹顶鹤电视剧制作中心、向海影视基地等项目前期工作。做大做强风雷网络科技等科技含量高、发展前景好的文化产业。

　　培育生态旅游品牌。规划建设总投资50亿元的24个旅游项目，发展生态旅游产业。加大宣传推介，提升旅游节庆活动。全年接待国内外游客246万人次，实现旅游收入24.8亿元，分别增长27%和28%。

　　抓好现代金融服务。实施金融创新拓展计划。搞好洮北信用联社改制。积极引进域外商业银行，力争五个县（市、区）都有村镇银行驻地服务。推进乡镇金融网点建设。加快中小企业担保公司招商重组。支持担保公司开展信誉评级。推进金融机构开发金融产品，改进金融服务方式，加大信贷投放力度。开展好“金融诚信年”活动。加强银企对接，解决中小企业融资难问题。

　　加强出口基地建设。坚持以通榆金禾、北方金塔等企业为龙头，做大出口企业群体，力争出口500万美元以上企业达到10户，出口1000万美元以上企业达到3户。继续抓好绿豆、葵花仁等出口基地建设，创建国家级绿豆出口基地，拓展国际市场。全市进出口总额达到1.26亿美元，增长15%。

　　（七）统筹城乡生态化发展，构建生态环境整治样板区。全力实施“环境立市”战略，打造天蓝、地绿、水清、气爽的白城。

　　推进造林绿化。继续开展“举市三年大造林”活动，力争完成造林40万亩，努力实现3年造林120万亩，夺取“三年大造林”全面胜利。强化通道绿化和城乡结合部绿化，打造精品绿化工程。积极争取国家“三北”五期百万亩人工林基地项目。扎实推进集体林权制度配套改革。

　　加强草原保护。充分利用国家草原生态保护政策，大力发展优质牧草，推广饲料作物种植。推行全面禁牧，新增草原围栏20万亩，人工种草10万亩，草场改良20万亩，建设好美丽草原。

　　抓好荒漠化治理。加强枸杞、沙枣、文冠果等10个示范项目5000亩基地建设。加快退化苇塘恢复工程，启动建设芦苇湿地自然保护区。推进防沙治沙示范区工程，治理荒漠化土地34.2万亩。

　　注重环境保护。扎实开展节能减排，确保县级污水处理厂稳定运行。单位GDP能源消耗率降低3.4%。抓好松花江流域防治规划实施。加快农村环境连片整治项目建设，实施畜禽养殖污染防治示范工程。

　　（八）加大招商引资力度，进一步扩大对外开放合作。坚持“走出去”与“请进来”并举，全年招商引资到位资金要达到540亿元，增长17.5%。

　　提高招商引资水平。坚持分区域、有重点开展组团招商、叩门招商和以商招商，盯住央企、突出民企、注重外企，抓项目、促合作，提高国内外大企业、战略投资者的引进率和落户率。开展“全员抓项目、全民抓招商”活动，鼓励支持更多的社会力量参与招商。组织好全国“两会”招商推介会、第八届“东博会”投资环境说明会等招商专题活动，推介项目、展示白城。

　　深入谋划产业项目。瞄准央企、民企结构调整方向，扩大合作领域，着力在清洁能源、油气化工、农产品加工、汽车配套、纺织医药、冶金建材、装备制造等七大优势产业，谋划一批投资额度大、带动能力强、产业关联度高的大项目。全市规划实施3000万元以上重点项目力争达到600个，储备项目300个以上。

　　完善项目建设机制。加强重点项目管理平台建设，对全市投资3000万元以上项目建立档案，实施项目形象的动态分析管理。全力推动签约项目立项、土地、环评等各项前期工作，制定针对性的配套政策和服务方案，确保合作项目尽快落地。落实领导包保项目责任制，对于特别重大项目，开辟“绿色通道”，实行“一项一议”、“一企一策”。

　　（九）努力保障和改善民生，加强和创新社会管理。围绕建设城乡统筹发展和谐区，加强以民生为重点的社会建设，全力办好利民惠民的实事、好事。

　　做好就业社会保障工作。全年新增城镇就业4.2万人，公益性岗位开发争取达到1.6万个，城镇“零就业”家庭保持动态为零，城镇登记失业率控制在4.6%以内。培育域外劳务输入基地110个以上，农村劳动力转移就业35.5万人。全面启动城镇居民养老保险试点。扩大新型农村养老保险试点覆盖面。积极推进医疗保险、工伤保险、生育保险等统筹。全市基本医疗保险覆盖率争取达到95%以上，在乡常住农业人口新农合参合率达到90%以上。

　　统筹发展各项社会事业。启动重大科技攻关工程，推进科技企业孵化基地建设。实施学前教育三年行动计划，改造和新建公办幼儿园17所。继续强化职教中心基础设施建设，努力提高职业教育发展水平。推动基础教育均衡发展。加强校车安全管理。继续推进和完善基层医疗改革，开展县级医院“收支两条线”和“药品零差率”试点。完成“规范化药房”建设。进一步稳定低生育水平，着力提高出生人口素质，人口自然增长率控制在4‰以内。积极应对人口老龄化趋势，促进幸福家庭建设。实施文化惠民工程。抓好精神文明创建活动。稳步推进事业单位改革。加强国防教育，持续广泛开展双拥工作。大力开展群众性文体活动，举办好白城市第五届田径运动会暨全民健身运动会。加强民族宗教工作，推进档案等事业实现新的发展。

　　集中力量办好民生实事。今年要全力办好十个方面民生实事。一是投资44.6亿元，全力实施保障性住房工程，改造棚户区218万平方米，建设廉租房20.2万平方米。二是投资7.4亿元，加快“暖房子”工程建设，改造供热管网199.8公里，既有建筑节能改造355万平方米。三是投资9000万元，建设15所农村标准化学校；投资3000万元，启动建设白城市素质教育实践基地。四是投资1.25亿元，扩建2.6万平方米社会精神病院病房楼，建设3500平方米军休服务中心，启动建设2万平方米白城市社会福利（养老）服务中心项目。五是投资2.3亿元，完成白城中心医院项目建设，加快推进白城中医院项目建设。六是投资8700万元，完成全民健身中心项目建设，改造白城市体育场。七是投资3000万元，完成7.5万户农村有线电视数字化整体改造，实现农村有线电视“村村通”、“户户通”。八是投资2500万元，解决5万农村人口饮水安全问题。九是投资1亿元，新建改建乡村公路和村村通等级公路200公里。十是实施贫困村整村推进计划，实现3万农村贫困人口脱贫。

　　全力维护社会和谐稳定。落实信访工作责任制，畅通群众诉求渠道，做好矛盾纠纷排查化解。妥善解决信访问题，力争全市个体访积案全部化解，集体访积案化解85%以上，当年新发生信访案件结案90%以上。深化平安白城创建活动，加强社会治安综合治理，完善社会治安巡逻防控体系。积极预防、依法打击各类重大刑事犯罪，提高人民群众安全感。“平安之声”开通率力争达到92%。推动社会管理重心下移，完善城乡社区管理服务体制，力争城镇社区公共服务中心全覆盖，推进“五有一创”工程实施。深化户籍制度改革，创新人口管理服务机制。严格落实安全生产责任制，加大安全生产隐患排查和专项治理力度，坚决遏制重特大事故发生，保持全市安全生产形势持续稳定。开展食品安全“大检查、大排查、大整改”集中行动和专项整治，严厉打击食品药品领域违法犯罪，确保人民群众饮食用药安全。加强应急管理，有效预防和处置突发事件。

　　三、进一步加强政府自身建设

　　发展的重任、人民的期待，对政府工作提出了更新更高的要求。人民政府，就要顺应人民的呼声、惠及人民的利益。我们必须提升推动发展、管理社会、服务群众的能力和水平，努力建设人民满意政府。

　　（一）强化依法行政，打造阳光政务，切实增强公信力。围绕建设法治型政府，推进依法治市进程，加大“六五”普法力度。严格按照法定权限和程序行使职权、履行职责，主动接受人大的工作监督、法律监督和政协民主监督，认真办理人大代表建议和政协委员提案。不断拓展政务公开的广度和深度。继续落实阳光政务“六项制度”，以实际成效取信于民。

　　（二）强化真抓实干，提升行政效能，切实增强执行力。深入开展“五讲”活动。坚持“干”字当头、“实”字当先，执行力要强、工作节奏要快、方式方法要活、工作效率要高、落实效果要好。对确定的目标不动摇，抓住工作不松手，一项一项地推进，形成加快发展谋大事、心无旁骛干实事、务实扎实干成事的氛围。要敢担当，发扬不畏艰难、不惧风险、不计得失的“三不”精神，敢于破解一切问题和矛盾。加强机关效能建设，全面推行政府绩效管理。进一步清理全市行政审批和收费项目，全力打造行政审批绿色通道。牢固树立“政府创优环境、企业创造财富”理念，实施“12345”服务工程，为企业、投资者和群众营造良好的服务环境。

　　（三）强化制度建设，规范行政行为，切实增强约束力。规范政府管理制度，对公共权力运行中涉及的重大事项决策、政策文件制定、会议议事、行政监督、依法行政等方面，从制度层面加以规范，推进政府工作制度化管理、流程化操作，形成比较完善的政府工作规范体系。加强反腐倡廉建设，认真落实廉政建设责任制，坚决纠正损害群众利益的不正之风，努力建设为民、务实、高效、廉洁政府。

　　各位代表：

　　回首过去历程，成就鼓舞人心；展望发展前景，目标催人奋进。让我们在市委的坚强领导下，开拓奋进，埋头苦干，扎实工作，为早日实现白城崛起振兴、人民生活更加美好而努力奋斗！
